# DiCART^TM^ device to measure capillary refill time: a validation study in patients with acute circulatory failure

**DOI:** 10.1007/s10877-025-01271-5

**Published:** 2025-02-26

**Authors:** Alexandre Descamps, Matthias Jacquet-Lagrèze, Thomas Aussal, Jean-Luc Fellahi, Martin Ruste

**Affiliations:** 1 Service d’anesthésie-réanimation, Hôpital cardiologique Louis Pradel, Hospices Civils de Lyon, Bron, France; 2 Faculté de médecine Lyon Est, Université Claude Bernard Lyon 1, Lyon, France; 3 CarMeN Laboratoire, Inserm UMR 1060, Université Claude Bernard Lyon 1, Lyon, France

**Keywords:** Capillary refill time, Acute circulatory failure, Validation, agreement, DiCART™

## Abstract

Capillary Refill Time (CRT) is a valuable metric to assess cutaneous perfusion. Its prognostic value in patients with acute circulatory failure has been reported as improved when the measurement is standardized. The DiCART™ device is a fully automated CRT measurement tool requiring validation. We conducted a comparative interventional single-center study including 25 patients with acute circulatory failure, to evaluate the agreement between CRT measured by an automated measurement device (CRT_DiCART_) and CRT measured clinically (CRT_CLIN_). CRT was measured on the fingertip, chest, and knee. Three measurements were performed at each location to obtain an average for each site. The measurements were conducted both clinically and using the DiCART™ device by two different operators, each blinded to the results. Agreement was determined using intraclass correlation coefficient (ICC) and Bland and Altman analysis. The ICC between CRT_CLIN_ and CRT_DiCART_ was 0.46 (95% Confidence Interval (CI) 0.32, 0.59) across all measurement sites; the mean bias was 0.23s (95% CI -0.17, 0.64), with upper Limit of Agreement (LoA) 2.77s (95% CI 2.44, 3.20) and lower LoA − 2.30s (-2.73, -1.97). Intra observer ICC was 0.85 (95% CI 0.74, 0.91) for CRT_CLIN_ and 0.43 (95% CI 0.15, 0.64) for CRT_DICART_. Inter observer ICC was 0.86 (95% CI 0.76, 0.92) for CRT_CLIN_ and was 0.41 (95% CI 0.14, 0,63) for CRT_DICART_. The DiCART™ device showed poor agreement with clinical CRT in patients with acute circulatory failure, which does not support its use in routine practice.

**Supplementary Information:**

The online version contains supplementary material available at 10.1007/s10877-025-01271-5.

## Introduction

The capillary refill time (CRT) is a dynamic clinical parameter of cutaneous perfusion that is used on a daily basis by emergency physicians, pediatricians, and intensivists. The measurement of this parameter has a prognostic value and is useful to guide the treatment in various contexts of acute circulatory insufficiency, such as sepsis [1] or dehydration states in children [2]. Its use is recommended by numerous scientific societies [3’^5^].

Nevertheless, the measurement of capillary refill time is subject to a number of limitations and remains a subjective and non-standardized evaluation. The reliability of this measurement may be influenced by various factors, including the location of the evaluation, the level and duration of the pressure applied and the training of the raters [6’^8^]. Furthermore, the precision of this method seems to be influenced by environmental factors such as temperature and brightness [9]. A survey among physicians reported a high rate of CRT measurement use, but through heterogeneous methods [10]; therefore, a better standardization would increase its accuracy in predicting death or adverse effects [11].

The DicarTech Company (Davézieux, France) is developing a medical device aiming to automatize the measurement of CRT. A clinical study demonstrated the safety and diagnostic efficacy of an initial prototype in healthy volunteers [12]. Furthermore, feedback resulted in the development of a second-generation prototype, which led to significant improvements of the device according to preliminary results [13]. The objective of this study was to assess the degree of concordance between the DiCART™ second-generation prototype device and clinically measured capillary refill time in patients presenting with acute circulatory failure.

## Methods

### Study design and settings

The study protocol was approved by an institutional review board (*Centre de Protection des Personnes Sud-Med 1*, 05/04/2022). Written informed consent was collected from each participant prior to their participation when their clinical condition allowed it. Otherwise, it was obtained from a family member. When the patient’s clinical condition improved subsequently, their consent was sought at the earliest opportunity. This study was conducted in the Cardiothoracic and Vascular Intensive Care Unit of the Louis Pradel University Hospital, *Hospices Civils de Lyon*, Lyon, France. The study was registered in ClinicalTrials.gov (NCT05847998, 02/16/2022).

### Participants

Patients aged of at least 18 years with acute circulatory failure were eligible for inclusion in the study. Acute circulatory failure was defined as a mean arterial pressure (MAP) lower than 70 mmHg or inotropic or vasopressor therapy to maintain a MAP greater than 65 mmHg, in conjunction with at least one indicator of tissue hypoperfusion, comprising mottling, hyperlactatemia greater than 2 mmol/L, oliguria, or confusion (defined with the Confusion Assessment Method [14]).

The exclusion criteria were the presence of skin lesions at the measurement site, current pregnancy or breastfeeding, lack of effective contraception in women with childbearing potential, absence of social security coverage, legal protection measures for patients, and participation in another interventional study.

### The DiCART™ device

**Fig. 1:**
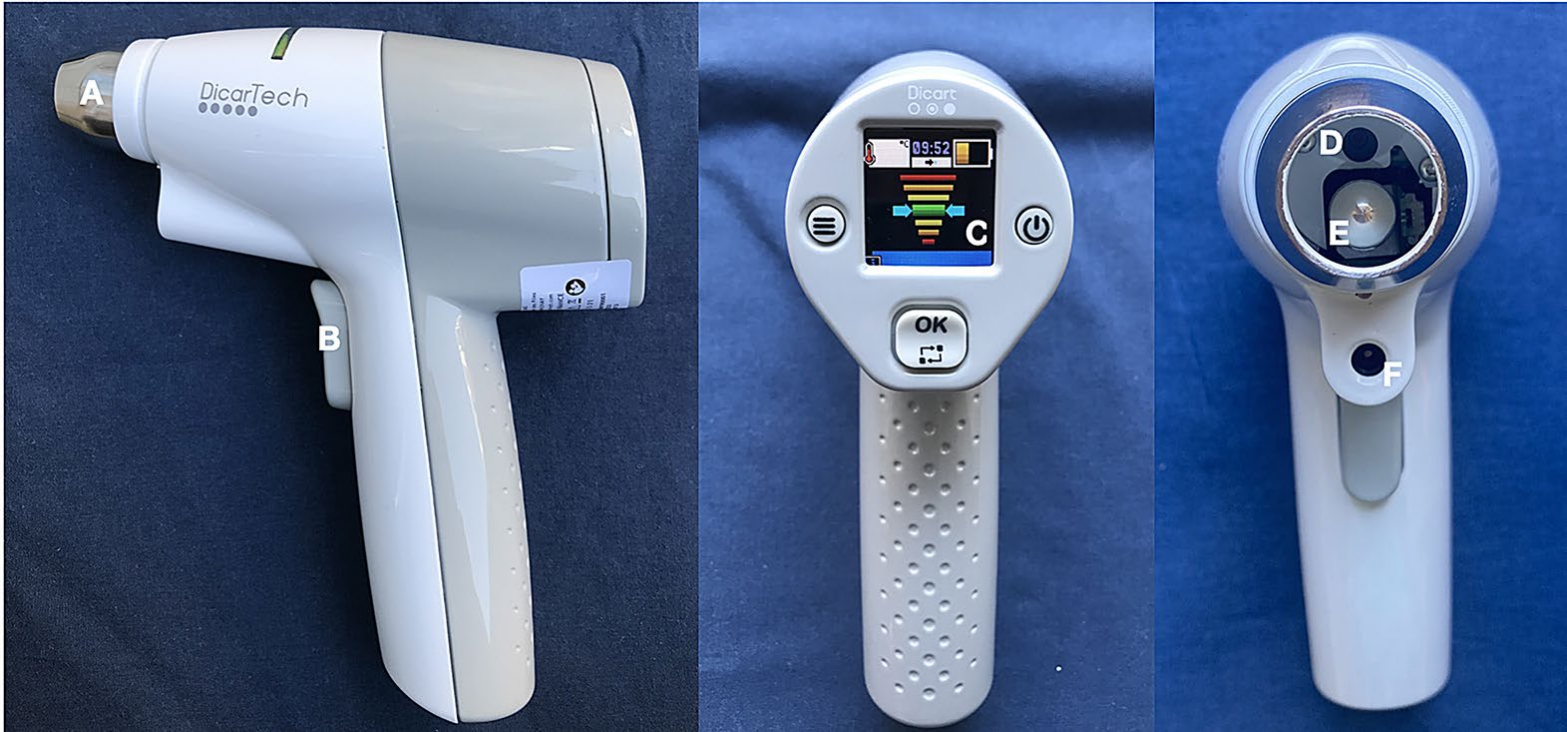
DiCART™ device. **A**: Spring connected to the pressure sensor; **B**: Start trigger; C: Screen displaying adynamic gauge of exerted pressure; **D**: Camera; **E**: Piston; **F**: Temperature sensor

The DiCART™ prototype is a fully automated device for CRT measurement (Fig. 1). The second-generation of the device has been designed and manufactured in accordance with a standardized process, enabling certification and industrial production. It has a compact design, with dimensions of 15 × 18 cm and a weight of 450 g, which allows its use with a single hand. It features a user interface comprising a 1.44-inch display, control buttons, and a joystick for navigation. It includes a temperature sensor for indicative surface temperature measurements. The device comprised a mechatronic system. The device is initially positioned on the patient’s skin for use. The measurement process is initiated through a trigger mechanism. Subsequently, the piston exerts a pressure on the skin for a period of five seconds, thereby inducing a blanching effect of the skin. The pressure applied by the piston is adjusted using a precision sensor and a calibrated spring system, thereby ensuring consistent force application. The sensors provide real-time feedback to maintain the expected pressure throughout the compression period. Subsequently, the piston retracts, and a high-precision camera captures Red Green Blue (RGB) images at 30 frames per second with a resolution of 1920 × 1080 pixels and a viewing angle of 64 × 48 degrees. The image acquisition process lasts for eight seconds. To ensure standardized lighting conditions, low-intensity pure white LEDs are used. The captured images are then processed by an embedded algorithm. This algorithm first converts the images from the RGB color space to the L*a*b color space and smooths the curves along the ‘a’ axis (red-green axis). It then calculates the averages of positive and negative gradients. Finally, the CRT is determined when the sum of positive gradients exceeds 50% of the total gradients.

### Study protocol

**Fig. 2:**
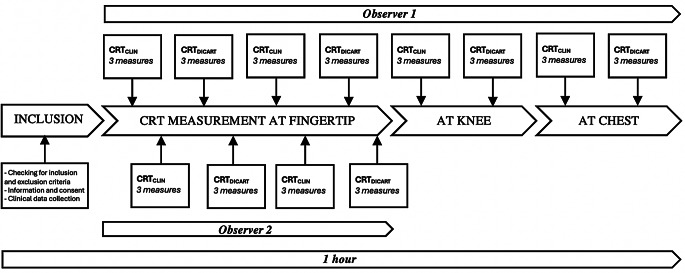
Experimental design of the study. CRT: Capillary refill time, CRT_CLIN_: Clinical measurement of CRT, CRT_DICART_: CRT measured with the DiCART™ automated device. Each measurement was performed 3 times to determine a mean CRT. Fingertip CRT assessments were carried out alternately by observer 1 and 2 in the shortest possible time

At baseline, the following data were collected for each patient: demographics, comprising age, sex, height, weight, body mass index, and phototype (Fitzpatrick Scale I to VI); medical history, comprising arterial hypertension, diabetes, ischemic cardiopathy, chronic obstructive pulmonary disease, peripheral arterial disease, and chronic kidney disease; clinical assessments, comprising shock mechanism, ventilation status, presence of confusion, urine output, mean arterial pressure, central venous pressure, heart rate, oxygen saturation, peripheral perfusion index [15], cardiac index, and mottling score [16]; medications, comprising administration of dobutamine, vasopressin, and norepinephrine; laboratory measurements, comprising plasmatic creatinine, arterial pH, arterial partial pressure of oxygen (PaO2), lactate, difference between venous and arterial partial pressure of carbon dioxide (pCO2 gap), transaminases, gamma-glutamyl transferase, alkaline phosphatase, bilirubin; and Sequential Organ Failure Assessment (SOFA) score [17]. Temperature measurements were taken for room temperature, central temperature (urinal or tympanic), and cutaneous temperature (measured with the DiCART™ device’s temperature sensor). Then, CRT were measured by two operators, both clinically and using the DiCART™ device, with each operator blinded to their own results. To estimate clinical CRT, the operator applied a firm pressure (characterized by the observer’s tip of the nail turning white) at the measurement site, for a duration of five seconds, which aligns with the compression time used by the DiCART™ device. Subsequently, the CRT was assessed using a chronometer. In order to be blinded to the result of the clinical CRT, the observer performing it gave a vocal signal (‘top’) at the moment of releasing the compression and another signal when he considered that the skin color had returned to its baseline state, allowing the second observer to start and stop the chronometer accurately. Each operator performed two series of three consecutive clinical measurements (CRT_CLIN_) and with the DiCART™ device (CRT_DiCART_) on the fingertip. Then, one operator performed a train of three measurements on the knee and on the chest (Fig. 2). CRT_CLIN_ and CRT_DiCART_ were carried out alternatively to enhance the comparability between the two methods. To finish, skin integrity was checked in the areas where the device was applied, and discomfort as well as pain were assessed using a numeric rating scale (range 0–10) when the patient was able to cooperate. At day 30, the following characteristics were collected: vital status and need for renal replacement therapy, length of stay in intensive care unit, duration of mechanical ventilation, and vasopressor support.

### Endpoints

The primary endpoint was the concordance between CRT measured clinically and using the DiCART™, assessed by the intraclass correlation coefficient (ICC). Secondary endpoints were: the diagnostic performance of the DiCART™ in detecting a CRT > 3 s established by the reference clinical test and expressed by the area under the receiver operating characteristic curve (ROC_AUC_); the inter and intra observer reproducibility of CRT_CLIN_ and CRT_DiCART_ (ICC and least significant change); and the clinical tolerance (skin lesions, pain, itching) of the DiCART™ device.

### Statistical analysis

The sample size calculation was based on the primary endpoint to detect an ICC of at least 0.6 with a power of 0.9 and an alpha risk of 0.05. We calculated a sample size of 23 patients, round to 25 [18]. The variable distribution was explored with the analysis of skewness and kurtosis, and with the Shapiro-Wilk test. No imputation was used for missing data (which are reported in Additional File, Table 1), variables with more than 25% of missing values were not analyzed. As DiCART™ devices are limited to estimate CRT ≤ 8 s, all the couple of measurements with a CRT_CLIN_ > 8 s were excluded from the analysis. For the whole analysis, 3 measurements were averaged to define CRT_CLIN_ and CRT_DiCART_. To describe the variability of the CRT_CLIN_ and CRT_DiCART_, the least significant change was calculated as previously described [19]. Agreement between CRT_CLIN_ and CRT_DiCART_ was estimated with ICC (model: two-way random effects; type: single rater; definition: consistency) [20] and Bland and Altman approach with confidence interval (CI) estimation by the MOVER approach to take into account for multiple observations per individual [21]. Thresholds for ICC interpretation defined a priori were based on the literature [20], and we defined acceptable limits of agreement (LoA) as ± 1 s as it is a threshold higher than the least significant changes previously described [22, 23] and that allows to discriminate patients with a normal from those with a high CRT. To analyze the intra and inter observer reproducibility of the two methods in the fingertip, we calculated the respective ICC between the two series of CRT measurements carried out by the same operator and between the measurements of the two operators. Receiver operating characteristic curves were constructed to evaluate the diagnostic performances of the DICART™ device to detect an abnormal clinical CRT on the fingertip, defined as CRT > 3 s. ROC_AUC_, sensitivity, specificity, and best threshold were determined, with 95% CI by bootstrap analysis with 2000 repetitions. Renal replacement therapy and vital status at day 30 were compared in high CRT_DiCART_ versus low, separated by the median value on the fingertip of CRT_DiCART_ in the present population, and compared using Fisher’s exact tests. Statistical analysis was performed using R software with the packages *pROC* and *SimplyAgree*. All tests were two-sided, and a P-value < 0.05 was considered statistically significant.

**Table 1:** Patients characteristics at baseline

*N* = 25	
Median age, years [25th − 75th percentile]	64 [60, 74]
Sex, female, n (%)	5 (20)
Mean Body Mass Index, kg/m^2^ (SD)	27.4 ± 3.9
Phototype, n (%)	
II	13 (52)
III	6 (24)
IV	6 (24)
Medical history, n (%)	
Arterial hypertension	9 (36)
Diabetes	4 (16)
Ischemic cardiopathy	10 (40)
Chronic obstructive pulmonary disease	2 (8)
Peripheral arterial disease	2 (8)
Chronic kidney disease	3 (12)
Shock mechanism, n (%)	
Cardiogenic	17 (68)
Septic	4 (16)
Hypovolemic	2 (8)
Non-septic vasoplegic	1 (4)
Mixed	1 (4)
Mean SOFA score ± SD	8 ± 4
Mechanical ventilation, n (%)	4 (16)
Confusion, n (%)	5 (13)
Median urinary output, ml/kg/h [25th − 75th percentile]	0.5 [0.3, 1.0]
Mean arterial pressure, mmHg ± SD	73 ± 9
Mean central venous pressure, mmHg ± SD	11 ± 6
Mean heart rate, /min ± SD	94 ± 17
Mean SpO2, % ± SD	95 ± 3
Median peripheral perfusion index, [25th − 75th percentile]	0.7 [0.2, 1.7]
Median mottling score, [25th − 75th percentile]	1 [1, 2]
Dobutamine administration, n (%)	12 (48)
Vasopressin administration, n (%)	2 (8)
Norepinephrine administration, n (%)	19 (76)
Median norepinephrine dosage, µg/kg/min [25th − 75th percentile]	0.13 [0.03, 0.24]
Median plasmatic creatinine, µmol/L [25th − 75th percentile]	124 [107, 196]
Mean arterial lactate, mmol/L ± SD	2.4 ± 1
Mean pCO2 gap, mmHg ± SD	6.2 ± 3.9
Mean room temperature, °C ± SD	22.7 ± 1.1
Mean patient central temperature, °C ± SD	37.2 ± 0.5
Median patient cutaneous, °C [25th − 75th percentile]	30.1 [29.2, 31.3]

SD: Standard Deviation, SOFA score: Sequential Organ Failure Assessment score, SpO2: blood oxygen saturation by pulse oximetry; pCO2 gap: difference between venous and arterial partial pressure of carbon dioxide. Patient’s central temperatures were either urinary or tympanic temperature

## Results

**Fig. 3:**
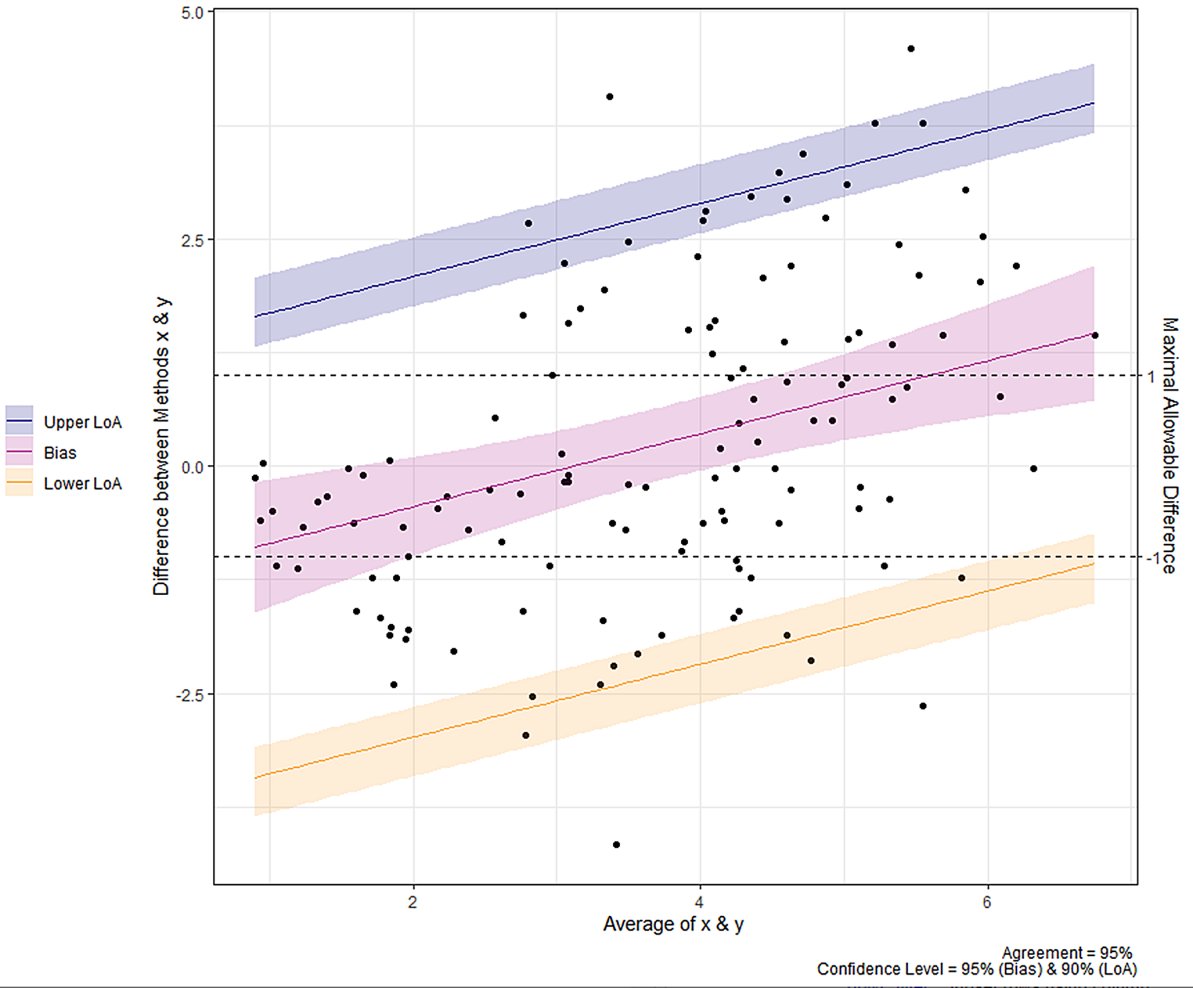
Bland and Altman plot assessing the agreement between the DiCART™ device and clinical measurement for capillary refill time (all measurement sites). *LoA: limit of agreement*,* represented with their 95% confidence interval; x: capillary refill time measured with clinical method; y: capillary refill time measure with DiCART™ method. Dashed lines represent the acceptable limits of agreement defined a priori in the study*

**Fig. 4:**
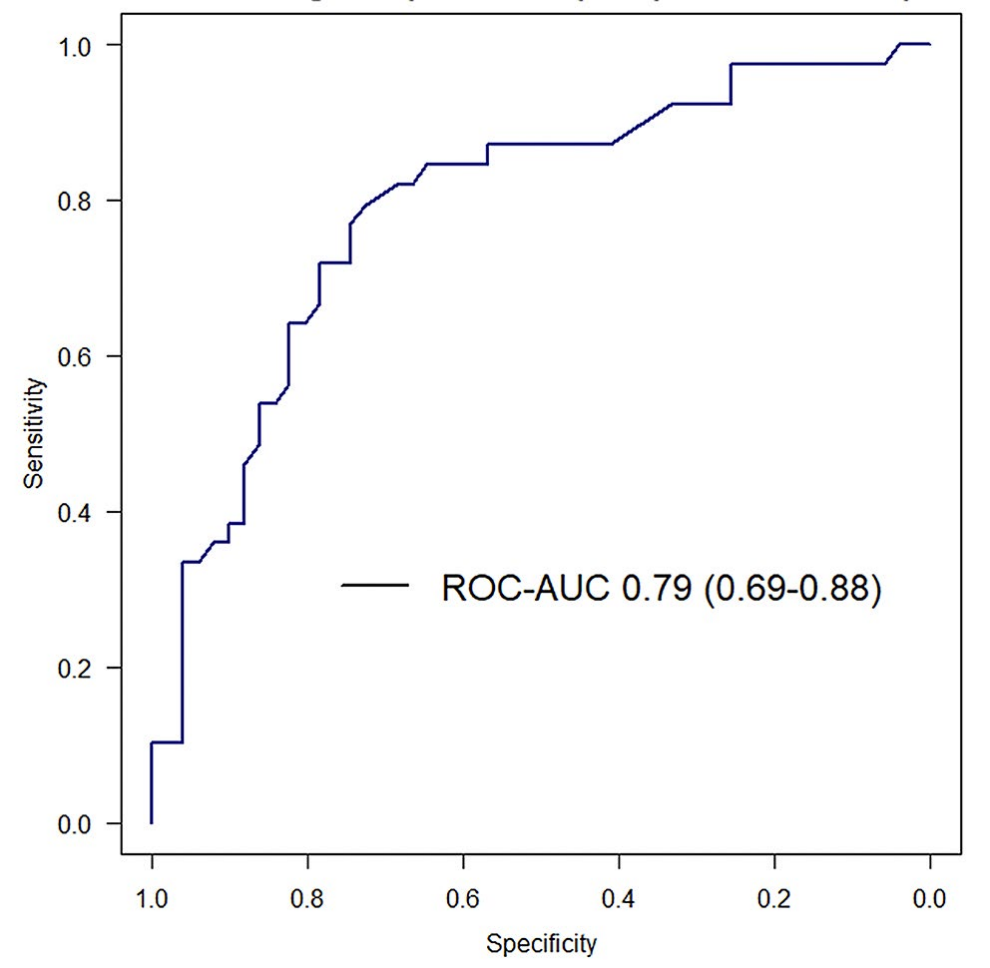
Receiver operating characteristic curve of the DiCART™ diagnostic performance to detect a capillary refill time higher than 3 s. *ROC-AUC: area under the receiver characteristic curve*,* with its 95% confidence interval*

Twenty-five patients were enrolled between April and October 2023. The study population was predominantly male (80%) and the median age was 64 [60, 74] years. The mechanism of shock was mainly cardiogenic (68%) and septic (16%). Mean SOFA score was 8 ± 4 (Table 1). The use of DiCART™ device did not encounter any mechanical issues and all measurements could be carried out. The median overall CRT_CLIN_ was 4.3s [2.4, 6.1]; 4.1s [1.6, 5.5] on the fingertip, 7.4s [4.2, 10.3] on the knee, and 3.9s [3.6, 4.4] on the chest. The median overall CRT_DiCART_ was 3.8s [2.7, 4.7]; 3.3s [2.5, 4.3] on the fingertip, 5.1s [3.5, 5.6] on the knee, and 4.5s [4.3, 4.8] on the chest. Twenty-two (15%) CRT paired measurements were excluded from the analysis due to an averaged CRT_CLIN_ > 8 s; 9 (9%) on the fingertip, 11 (44%) on the knee, and 2 (8%), on the chest. The ICC between CRT_DiCART_ and CRT_CLIN_ was 0.46 (95% CI 0.32, 0.59) overall, 0.5 (95% CI 0.32, 0.59) for the fingertip, 0.12 (95% CI -0.42, 0.60) for the knee, and 0.05 (95% CI -0.36, 0.45) for the chest (Table 2). Bland and Altman analysis is reported in Fig. 3; the mean bias was 0.23s (95% CI -0.17, 0.64), with upper LoA 2.77s (95% CI 2.44, 3.20) and lower LoA − 2.30s (-2.73, -1.97; Table 2). LoA calculation were corrected for proportional bias (Additional File, Fig. 1). Diagnostic performance of the DiCART™ to detect a CRT > 3 s had a ROC_AUC_ of 0.79 (95% CI 0.69, 0.88), with a sensitivity of 79 (95% CI 67, 90) %, a specificity of 76% (95% CI 63, 90) %, a positive predictive value of 71% (95% CI 62, 82), and a negative predictive value of 83% (95% CI 74, 91) for a best threshold of 3.1s (95% CI 2.8, 3.3; Fig. 4). Intra observer ICC on fingertip was 0.85 (95% CI 0.74, 0.91) for CRT_CLIN_ and 0.43 (95% CI 0.15, 0.64) for CRT_DICART_. Inter observer ICC was 0.86 (95% CI 0.76, 0.92) for CRT_CLIN_ and was 0.41 (95% CI 0.14, 0,63) for CRT_DICART_. CRT measurement variability was significantly lower for CRT_CLIN_ compared to CRT_DiCART_; least significant change 31% (95% CI 28, 35) *versus* 65% (95% CI 59, 72), p value < 0.001. No skin lesion was observed at the different measurement sites. All patients reported no discomfort or pain (median numeric rating scale value 0 [0, 0]). There was no significant difference regarding the need for renal replacement and vital status at day 30 between patients with a CRT_DiCART_ > 3.3s compared to the others (Table 3).

**Table 2:** Agreement analysis

Intra class correlation coefficients (95% CI)	
Overall	0.46 (0.32, 0.59)
Fingertip	0.50 (0.32, 0.64)
Knee	0.12 (-0.42, 0.60)
Chest Sternum	0.05 (-0.36, 0.45)
**All sites Bland and Altman analysis** (90% CI)	
Mean Bias, s	0.23 (-0.17, 0.64)
Mean Upper limit of agreement, s	2.77 (2.44, 3.20)
Mean Lower limit of agreement, s	− 2.30 (-2.73, -1.97)

**Table 3:** 30-day outcomes based on high or low value of capillary refill time measured with the *DiCART™* device

Variables	DiCART_LOW_ (*n* = 12)	DiCART_HIGH_ (*n* = 13)	*p* value
RRT status, n (%)			
No	10 (83.3)	10 (76.9)	
Current	1 (8.3)	0 (0)	0. 375
Withdrawal	1 (8.3)	3 (23.1)	
Vital status, n (%)			
Deceased	3 (25.0)	3 (23.1)	1. 000
Alive	9 (75.0)	10 (76.9)	

RRT: Renal Replacement Therapy, No: RRT was not needed, Current: RRT is ongoing, Withdrawal: RRT is weaned off

Capillary refill time values used for the analysis were the average of the means of the two observers’ first measurement series on fingertip. DiCART_LOW_: Means of capillary refill time measured with the DiCART™ device < 3.3 s. DiCART_HIGH_: Means of capillary refill time measured with the DiCART™ device > 3.3 s

## Discussion

The present study demonstrated a poor agreement between CRT_CLIN_ and CRT_DiCART_ in patients with acute circulatory failure. Furthermore, the device exhibited lower intra and interobserver reproducibility compared to the clinically measured CRT.

This study provided several insights into the current DiCART™ prototype. Firstly, the agreement between clinical CRT and CRT measured using the DiCART™ device was poor, with an ICC lower than 0.5. The Bland and Altman analysis also suggested that the DiCART™ device may not provide reliable measurements (i.e. consistent with the clinical method). The LoA were significantly greater than the a priori defined acceptable limits of -1 and + 1 s, indicating a high variability in the differences between the two methods. The agreement was reasonable when the difference was within one second to distinguish a normal CRT from an abnormal one with a 3-second threshold, which is a good predictor of poor outcome in critically ill patients [24]. The present results highlighted a systematic bias, using the DiCART™ method, which tended to record greater CRT compared to the clinical method. Additionally, there was a proportional bias, with a noticeable trend toward increased differences in measurements with greater ranges. In the validation study of the first DiCART™ prototype in healthy volunteers, concordance was also reported as poor, within the same range as herein [12]. The authors identified the pressure performed on the skin by the crown of the device, which appeared to alter the capillary refill time, as a significant problem. To develop the second-generation prototype, a spring connected to a pressure sensor was added to the device, allowing the user to control the pressure performed on the skin. However, this addition did not improve the reliability of the device.

On the other hand, the previous validation study demonstrated the device’s ability to diagnose tissue hypoperfusion during an upper limb arterial occlusion test, with both sensitivity and specificity exceeding 90% [12]. In the present study, these results were not confirmed in patients with acute circulatory failure. An explanation could be that a vascular occlusion test is a caricature of cutaneous ischemia whereas cutaneous microcirculatory impairment in patients with acute circulatory failure can be much more heterogeneous. While reporting significant improvements of the device, the previous bench and in silico study has shown a decrease in reliability and reproducibility when random perturbations (brightness, contrast, saturation) were applied to videos originating from healthy volunteers, also highlighting some limitations of the device’s algorithm [13].

Several studies have assessed CRT measurement devices that used various technologies. While some of them have yielded promising results, they have been conducted mostly on healthy volunteers under experimental conditions, with a particular focus on cutaneous cooling [25’^27^]. Shavit et al. measured CRT using a digital camera and custom software that compares each frame after the release of a 5-second fingertip compression with the initial pre-compression frame, calculating CRT when an exact color match is achieved [28]. In children suffering from gastroenteritis, the use of a digital camera with custom software to analyze cutaneous recoloration had shown excellent diagnostic performance to detect significant dehydration (AUC 0.99) as well as high intra observer reliability (ICC 0.99), but the application of the compression in the skin was not controlled by the device, potentially leading to variability. The Digital Capillary Refill (DCR) device, developed by ProMedix Inc. (Portland, OR, USA) is a compact handled prototype that consists in a finger sensor applied on the fingertip. The CRT is measured using light reflectance technology following manual compression. Correlation coefficient range between the device estimated CRT and the clinically measured CRT ranged from 0.69 to 0.91 [29, 30], and a prolonged CRT estimated by the device was associated with the diagnosis of sepsis [31]. However, these studies were not designed to evaluate the agreement of the methods nor the diagnostic performance of the device.

One of the difficulties in studying CRT is the absence of a well-defined gold standard method for its measurement. Numerous techniques to clinically measure CRT were described in the literature [32]. Measuring CRT after a 5-second compression using a stopwatch and averaging the results of three measurements allowed to achieve herein good inter and intra observer agreement. Similar concordance results have already been published, such as in patients with septic shock for whom a similar inter observer agreement was already observed using a 15-second time of compression on the fingertip, averaged over two measurements [33, 34]. Conversely, using a single, non-timed measurement, performed by a heterogeneous population of investigators comprising nurses, medical students, residents, and senior physicians led to only a moderate inter observer agreement [7].

The present study has however limitations. The generalizability is limited by the single-center design the small sample size of the study that included a high proportion of patients who experienced cardiogenic shock. One can argue that assessing a new version of the DiCART™ was premature despite the encouraging preliminary results; the modifications performed on the device were likely insufficient, focusing more on the structure. Another issue inherent to the metric assessed (CRT) is the difficulties to determine a reference method. However, the method used herein for CRT_CLIN_ was supported by a recent meta-analysis [11], and the variability of the measure estimated by the least significant change is similar to previous studies [22, 23]. By analogy with the vascular occlusion test, the repetition of CRT assessment in the present study may have introduced a systematic bias in CRT estimation. A recent study showing that the interval between different CRT assessments does not affect the CRT value does not support this assumption [35]. However, randomization of the starting technique in the protocol would have further mitigated this potential bias.

Although the results provided by the DiCART™ were not sufficient, the study has been conducted with: (1) a strict experimental protocol with repeated measurements on various sites and blinding of the observers; (2) a strict statistical analysis to assess the agreement according to the suggestions of a dedicated recent review [36]; and (3) in a population in whom CRT assessment could be challenging but useful as a monitoring tool to guide resuscitation strategies [37] or to help to assess prognosis [38].

## Conclusion

The DiCART™ device showed poor agreement with clinical CRT in patients with acute circulatory failure, which does not support its use in routine practice.

## Electronic supplementary material

Below is the link to the electronic supplementary material.

Supplementary Material 1

## Data Availability

Data is available from the corresponding author upon reasonable request.

## Declarations

### Take home message

DiCART™ is a full automatized handled device to assess capillary refill time. Its agreement with clinical evaluation of capillary refill time is poor. Its reproducibility is lower than clinical evaluation of capillary refill time.

## References

[1] Singer M, Deutschman CS, Seymour CW, Shankar-Hari M, Annane D, Bauer M, Bellomo R, Bernard GR, Chiche J-D, Coopersmith CM, Hotchkiss RS, Levy MM, Marshall JC, Martin GS, Opal SM, Rubenfeld GD, van der Poll T, Vincent J-L, Angus DC. The Third International Consensus definitions for Sepsis and septic shock (Sepsis-3). JAMA. 2016;315:801–10. 10.1001/jama.2016.0287. 26903338 10.1001/jama.2016.0287 PMC4968574

[2] Fleming S, Gill P, Jones C, Taylor JA, Van den Bruel A, Heneghan C, Roberts N, Thompson M. The diagnostic value of Capillary Refill Time for detecting Serious illness in children: a systematic review and Meta-analysis. PLoS ONE. 2015;10:e0138155. 10.1371/journal.pone.0138155. 26375953 10.1371/journal.pone.0138155 PMC4573516

[3] Cecconi M, De Backer D, Antonelli M, Beale R, Bakker J, Hofer C, Jaeschke R, Mebazaa A, Pinsky MR, Teboul JL, Vincent JL, Rhodes A. Consensus on circulatory shock and hemodynamic monitoring. Task force of the European Society of Intensive Care Medicine. Intensive Care Med. 2014;40:1795–815. 10.1007/s00134-014-3525-z. 25392034 10.1007/s00134-014-3525-z PMC4239778

[4] Davis AL, Carcillo JA, Aneja RK, Deymann AJ, Lin JC, Nguyen TC, Okhuysen-Cawley RS, Relvas MS, Rozenfeld RA, Skippen PW, Stojadinovic BJ, Williams EA, Yeh TS, Balamuth F, Brierley J, De Caen AR, Cheifetz IM, Choong K, Conway E, Cornell T, Doctor A, Dugas M-A, Feldman JD, Fitzgerald JC, Flori HR, Fortenberry JD, Graciano AL, Greenwald BM, Hall MW, Han YY, Hernan LJ, Irazuzta JE, Iselin E, Van Der Jagt EW, Jeffries HE, Kache S, Katyal C, Kissoon N, Tex), Kon AA, Kutko MC, MacLaren G, Maul T, Mehta R, Odetola F, Parbuoni K, Paul R, Peters MJ, Ranjit S, Reuter-Rice KE, Schnitzler EJ, Scott HF, Torres A, Weingarten-Abrams J, Weiss SL, Zimmerman JJ, Zuckerberg AL. American College of Critical Care Medicine Clinical Practice Parameters for hemodynamic support of Pediatric and neonatal septic shock. Crit Care Med. 2017;45:1061–93. 10.1097/CCM.0000000000002425. 10.1097/CCM.0000000000002425 28509730

[5] Evans L, Rhodes A, Alhazzani W, Antonelli M, Coopersmith CM, French C, Machado FR, Mcintyre L, Ostermann M, Prescott HC, Schorr C, Simpson S, Wiersinga WJ, Alshamsi F, Angus DC, Arabi Y, Azevedo L, Beale R, Beilman G, Belley-Cote E, Burry L, Cecconi M, Centofanti J, Coz Yataco A, De Waele J, Dellinger RP, Doi K, Du B, Estenssoro E, Ferrer R, Gomersall C, Hodgson C, Møller MH, Iwashyna T, Jacob S, Kleinpell R, Klompas M, Koh Y, Kumar A, Kwizera A, Lobo S, Masur H, McGloughlin S, Mehta S, Mehta Y, Mer M, Nunnally M, Oczkowski S, Osborn T, Papathanassoglou E, Perner A, Puskarich M, Roberts J, Schweickert W, Seckel M, Sevransky J, Sprung CL, Welte T, Zimmerman J, Levy M. Surviving sepsis campaign: international guidelines for management of sepsis and septic shock 2021. Intensive Care Med. 2021;47:1181–247. 10.1007/s00134-021-06506-y. 34599691 10.1007/s00134-021-06506-y PMC8486643

[6] Kawaguchi R, Nakada T, Oshima T, Shinozaki M, Nakaguchi T, Haneishi H, Oda S. Optimal pressing strength and time for capillary refilling time. Crit Care. 2019;23:4. 10.1186/s13054-018-2295-3. 30621748 10.1186/s13054-018-2295-3 PMC6323707

[7] Alsma J, Van Saase JLCM, Nanayakkara PWB, Schouten WEMI, Baten A, Bauer MP, Holleman F, Ligtenberg JJM, Stassen PM, Kaasjager KHAH, Haak HR, Bosch FH, Schuit SCE, Arends JJ, Buunk G, Veldman BJA, Ammerlaan HSM, Sankatsing SUC, Jacobs EMG, Van Bemmel T, Ruiter R, Bots EMT, Reuters RA, Carels G, Diepeveen SHA, Heitz AFN, Van Hien TT, Keurlings PAJ, Barnhard R, Schreurs RHP, Ter Avest E, Brink HS, Van Kinschot CMJ, Van Der Hoeven N, Van Der Zijden MA, Hageman IMG, Roeleveld TC, Klomp CMC, Dekker D, Blom A, Wesselius HM, Van Bemmel MM, De Jong B, Hillen J, Langbroek G-B, De Bie S. The Power of Flash Mob Research. Chest. 2017;151:1106–13. 10.1016/j.chest.2016.11.035. 27940191 10.1016/j.chest.2016.11.035

[8] Shinozaki K, Jacobson LS, Saeki K, Kobayashi N, Weisner S, Falotico JM, Li T, Kim J, Lampe JW, Becker LB. Does training level affect the accuracy of visual assessment of capillary refill time? Crit Care. 2019;23:157. 10.1186/s13054-019-2444-3. 31060576 10.1186/s13054-019-2444-3 PMC6501297

[9] Brown LH, Prasad NH, Whitley TW. Adverse lighting condition effects on the assessment of capillary refill. Am J Emerg Med. 1994;12:46–7. 10.1016/0735-6757(94)90196-1. 8285971 10.1016/0735-6757(94)90196-1

[10] Jacquet-Lagrèze M, Wiart C, Schweizer R, Didier L, Ruste M, Coutrot M, Legrand M, Baudin F, Javouhey E, Dépret F, Fellahi J-L. Capillary refill time for the management of acute circulatory failure: a survey among pediatric and adult intensivists. BMC Emerg Med. 2022;22:131. 10.1186/s12873-022-00681-x. 35850662 10.1186/s12873-022-00681-x PMC9290243

[11] Jacquet-Lagrèze M, Pernollet A, Kattan E, Ait-Oufella H, Chesnel D, Ruste M, Schweizer R, Allaouchiche B, Hernandez G, Fellahi J-L. Prognostic value of capillary refill time in adult patients: a systematic review with meta-analysis. Crit Care. 2023;27:473. 10.1186/s13054-023-04751-9. 38042855 10.1186/s13054-023-04751-9 PMC10693708

[12] Ruste M, Cazenave L, Tardif M, Saint-Jean C, Fellahi J-L, Lagrèze MJ. Measurement of capillary refill time with a handheld prototype device: a comparative validation study in healthy volunteers. J Clin Monit Comput. 2022;36:1271–8. 10.1007/s10877-021-00757-2. 34550528 10.1007/s10877-021-00757-2

[13] Jacquet-Lagrèze M, Saint-Jean C, Bouët T, Reynaud S, Ruste M, Fellahi J-L. Reliability and reproducibility of the DICART device to assess capillary refill time: a bench and in-silico study. J Clin Monit Comput. 2023;37:1409–12. 10.1007/s10877-023-01027-z. 37199880 10.1007/s10877-023-01027-z

[14] Inouye SK, van Dyck CH, Alessi CA, Balkin S, Siegal AP, Horwitz RI. Clarifying confusion: the confusion assessment method. A new method for detection of delirium. Ann Intern Med. 1990;113:941–8. 10.7326/0003-4819-113-12-941. 2240918 10.7326/0003-4819-113-12-941

[15] Coutrot M, Dudoignon E, Joachim J, Gayat E, Vallée F, Dépret F. Perfusion index: physical principles, physiological meanings and clinical implications in anaesthesia and critical care. Anaesth Crit Care Pain Med. 2021;40:100964. 10.1016/j.accpm.2021.100964. 34687923 10.1016/j.accpm.2021.100964

[16] Ait-Oufella H, Lemoinne S, Boelle PY, Galbois A, Baudel JL, Lemant J, Joffre J, Margetis D, Guidet B, Maury E, Offenstadt G. Mottling score predicts survival in septic shock. Intensive Care Med. 2011;37:801–7. 10.1007/s00134-011-2163-y. 21373821 10.1007/s00134-011-2163-y

[17] Vincent JL, Moreno R, Takala J, Willatts S, De Mendonça A, Bruining H, Reinhart CK, Suter PM, Thijs LG. The SOFA (Sepsis-related Organ failure Assessment) score to describe organ dysfunction/failure. On behalf of the Working Group on Sepsis-related problems of the European Society of Intensive Care Medicine. Intensive Care Med. 1996;22:707–10. 10.1007/BF01709751. 8844239 10.1007/BF01709751

[18] Zou GY. Sample size formulas for estimating intraclass correlation coefficients with precision and assurance. Stat Med. 2012;31:3972–81. 10.1002/sim.5466. 22764084 10.1002/sim.5466

[19] Cecconi M, Rhodes A, Poloniecki J, Della Rocca G, Grounds RM. Bench-to-bedside review: the importance of the precision of the reference technique in method comparison studies– with specific reference to the measurement of cardiac output. Crit Care. 2009;13:201. 10.1186/cc7129. 19183431 10.1186/cc7129 PMC2688094

[20] Koo TK, Li MY. A Guideline of selecting and reporting Intraclass correlation coefficients for Reliability Research. J Chiropr Med. 2016;15:155–63. 10.1016/j.jcm.2016.02.012. 27330520 10.1016/j.jcm.2016.02.012 PMC4913118

[21] Zou GY. Confidence interval estimation for the bland-Altman limits of agreement with multiple observations per individual. Stat Methods Med Res. 2013;22:630–42. 10.1177/0962280211402548. 21705434 10.1177/0962280211402548

[22] Jacquet-Lagrèze M, Bouhamri N, Portran P, Schweizer R, Baudin F, Lilot M, Fornier W, Fellahi J-L. Capillary refill time variation induced by passive leg raising predicts capillary refill time response to volume expansion. Crit Care. 2019;23:281. 10.1186/s13054-019-2560-0. 31420052 10.1186/s13054-019-2560-0 PMC6697974

[23] Fage N, Moretto F, Rosalba D, Shi R, Lai C, Teboul J-L, Monnet X. Effect on capillary refill time of volume expansion and increase of the norepinephrine dose in patients with septic shock. Crit Care. 2023;27:429. 10.1186/s13054-023-04714-0. 37932812 10.1186/s13054-023-04714-0 PMC10629142

[24] Sebat C, Vandegrift MA, Oldroyd S, Kramer A, Sebat F. Capillary refill time as part of an early warning score for rapid response team activation is an independent predictor of outcomes. Resuscitation. 2020;153:105–10. 10.1016/j.resuscitation.2020.05.044. 32504768 10.1016/j.resuscitation.2020.05.044

[25] John RT, Henricson J, Nilsson GE, Wilhelms D, Anderson CD. Reflectance spectroscopy: to shed new light on the capillary refill test. J Biophotonics. 2018;11:e201700043. 10.1002/jbio.201700043. 10.1002/jbio.201700043 28544641

[26] Blaxter LL, Morris DE, Crowe JA, Henry C, Hill S, Sharkey D, Vyas H, Hayes-Gill BR. An automated quasi-continuous capillary refill timing device. Physiol Meas. 2016;37:83–99. 10.1088/0967-3334/37/1/83. 26642080 10.1088/0967-3334/37/1/83 PMC4770525

[27] Shinozaki K, Saeki K, Jacobson LS, Falotico JM, Li T, Hirahara H, Horie K, Kobayashi N, Weisner S, Lampe JW, Becker LB. Evaluation of accuracy of capillary refill index with pneumatic fingertip compression. J Clin Monit Comput. 2021;35:135–45. 10.1007/s10877-019-00454-1. 31916222 10.1007/s10877-019-00454-1

[28] Shavit I, Brant R, Nijssen-Jordan C, Galbraith R, Johnson DW. A novel imaging technique to measure capillary-refill time: improving diagnostic accuracy for dehydration in young children with gastroenteritis. Pediatrics. 2006;118:2402–8. 10.1542/peds.2006-1108. 17142525 10.1542/peds.2006-1108

[29] Gillespie J, Hansen M, Samatham R, Baker SD, Filer S, Sheridan DC. Capillary Refill Technology to enhance the Accuracy of Peripheral perfusion evaluation in Sepsis. J Intensive Care Med. 2022;37:1159–64. 10.1177/08850666221087685. 35306923 10.1177/08850666221087685

[30] Sheridan DC, Cloutier RL, Samatham R, Hansen ML. Point-Of-Care Capillary Refill Technology improves accuracy of Peripheral Perfusion Assessment. Front Med (Lausanne). 2021;8:694241. 10.3389/fmed.2021.694241. 34368191 10.3389/fmed.2021.694241 PMC8339369

[31] Hansen M, Gillespie J, Riddick T, Samatham R, Baker S, Filer S, Xin H, Sheridan D. Evaluation of electronic measurement of capillary refill for Sepsis screening at ED triage. Am J Emerg Med. 2023;70:61–5. 10.1016/j.ajem.2023.05.009. 37201452 10.1016/j.ajem.2023.05.009

[32] Huang W, Huang Y, Ke L, Hu C, Chen P, Hu B. Perspectives for capillary refill time in clinical practice for sepsis. Intensive Crit Care Nurs. 2024;84:103743. 10.1016/j.iccn.2024.103743. 38896965 10.1016/j.iccn.2024.103743

[33] Ait-Oufella H, Bige N, Boelle PY, Pichereau C, Alves M, Bertinchamp R, Baudel JL, Galbois A, Maury E, Guidet B. Capillary refill time exploration during septic shock. Intensive Care Med. 2014;40:958–64. 10.1007/s00134-014-3326-4. 24811942 10.1007/s00134-014-3326-4

[34] Raia L, Gabarre P, Bonny V, Urbina T, Missri L, Boelle P-Y, Baudel J-L, Guidet B, Maury E, Joffre J, Ait-Oufella H. Kinetics of capillary refill time after fluid challenge. Ann Intensive Care. 2022;12:74. 10.1186/s13613-022-01049-x. 35962860 10.1186/s13613-022-01049-x PMC9375797

[35] Meyer F, Henricson J, Anderson CD, Wilhelms DB. The effect of repeated Capillary Refill tests on the cutaneous microcirculation. J Biophotonics. 2024;17:e202400098. 10.1002/jbio.202400098. 39227989 10.1002/jbio.202400098

[36] Abu-Arafeh A, Jordan H, Drummond G. Reporting of method comparison studies: a review of advice, an assessment of current practice, and specific suggestions for future reports. Br J Anaesth. 2016;117:569–75. 10.1093/bja/aew320. 27799171 10.1093/bja/aew320

[37] Hernández G, Ospina-Tascón GA, Damiani LP, Estenssoro E, Dubin A, Hurtado J, Friedman G, Castro R, Alegría L, Teboul J-L, Cecconi M, Ferri G, Jibaja M, Pairumani R, Fernández P, Barahona D, Granda-Luna V, Cavalcanti AB, Bakker J, for the ANDROMEDA-SHOCK Investigators and the Latin America Intensive Care Network (LIVEN). Effect of a Resuscitation Strategy Targeting Peripheral Perfusion Status vs serum lactate levels on 28-Day mortality among patients with septic shock: the ANDROMEDA-SHOCK Randomized Clinical Trial. JAMA. 2019;321:654. 10.1001/jama.2019.0071. 30772908 10.1001/jama.2019.0071 PMC6439620

[38] Merdji H, Curtiaud A, Aheto A, Studer A, Harjola V-P, Monnier A, Duarte K, Girerd N, Kibler M, Ait-Oufella H, Helms J, Mebazaa A, Levy B, Kimmoun A, Meziani F. Performance of early Capillary Refill Time Measurement on outcomes in cardiogenic shock: an observational, prospective multicentric study. Am J Respir Crit Care Med. 2022;206:1230–8. 10.1164/rccm.202204-0687OC. 35849736 10.1164/rccm.202204-0687OC

