# Supplementary material for: DiCARTTM device to measure capillary refill time: a validation study in patients with acute circulatory failure
Source: J Clin Monit Comput. 2025 Feb 26;39(5):831–40. doi: 10.1007/s10877-025-01271-5 (PMC12474624; doi:10.1007/s10877-025-01271-5)

**Additional file:**

Table 1: Missing values

| **Variables (N)** | **Count (%)** |
| --- | --- |
| Inclusion (25) | 0 (0) |
| Age (25) | 0 (0) |
| Sex (25) | 0 (0) |
| Height (25) | 0 (0) |
| Weight (25) | 0 (0) |
| Phototype (25) | 0 (0) |
| Arterial hypertension (25) | 0 (0) |
| Diabetes (25) | 0 (0) |
| Ischemic cardiopathy (25) | 0 (0) |
| Chronic obstructive pulmonary disease (25) | 0 (0) |
| Peripheral arterial disease (25) | 0 (0) |
| Chronic kidney disease (25) | 0 (0) |
| Shock mechanism (25) | 0 (0) |
| Sequential organ failure assessment score (25) | 0 (0) |
| Ventilation status (25) | 0 (0) |
| Confusion (25) | 2 (8) |
| Urine output (25) | 0 (0) |
| Mean arterial pressure (25) | 0 (0) |
| Central venous pressure (25) | 2 (8) |
| Heart rate (25) | 0 (0) |
| Oxygen saturation (25) | 1 (4) |
| Peripheral perfusion index (25) | 1 (4) |
| Cardiac index (25) | 11 (44) |
| Mottling score (25) | 0 (0) |
| Dobutamine administration (25) | 0 (0) |
| Dobutamine dosage (25) | 0 (0) |
| Vasopressin administration (25) | 0 (0) |
| Vasopressin dosage (25) | 0 (0) |
| Norepinephrine administration (25) | 0 (0) |
| Norepinephrine dosage (25) | 0 (0) |
| Plasmatic creatinine (25) | 0 (0) |
| pH (25) | 0 (0) |
| Arterial lactate (25) | 0 (0) |
| Arterial partial pressure of oxygen (25) | 0 (0) |
| Arterial partial pressure of carbon dioxide (25) | 0 (0) |
| Venous partial pressure of carbon dioxide (25) | 4 (16) |
| Aspartate aminotransferase (25) | 7 (28) |
| Alanine Aminotransferase (25) | 7 (28) |
| Gamma-glutamyl transferase (25) | 13 (52) |
| Bilirubin (25) | 13 (52) |
| Alkaline phosphatase (25) | 12 (48) |
| Room temperature (25) | 0 (0) |
| Patient cutaneous temperature (25) | 3 (12) |
| Patient central temperature (25) | 0 (0) |
| Device application’s pain numeric rating scale (25) | 4 (16) |
| Cutaneous lesion (25) | 0 (0) |
| 30-days vital status (25) | 0 (0) |
| 30-days mechanical ventilation status (25) | 0 (0) |
| 30-days renal replacement therapy (25) | 0 (0) |
| 30-days amine support duration (25) | 0 (0) |
| CRT_CLIN_ on fingertip, first measure (91) | 0 (0) |
| CRT_CLIN_ on fingertip, second measure (91) | 0 (0) |
| CRT_CLIN on_ fingertip, third measure (91) | 0 (0) |
| CRT_CLIN on_ fingertip, mean (91) | 0 (0) |
| CRT_DiCART_ on fingertip, first measure (91) | 1 (1.1) |
| CRT_DiCART_ on fingertip, second measure (91) | 1 (1.1) |
| CRT_DiCART_ on fingertip, third measure (91) | 1 (1.1) |
| CRT_DiCART_ on fingertip, mean (91) | 1 (1.1) |
| CRT_CLIN_ on knee, first measure (14) | 0 (0) |
| CRT_CLIN_ on knee, second measure (14) | 0 (0) |
| CRT_CLIN on_ knee, third measure (14) | 0 (0) |
| CRT_CLIN on_ knee, mean (14) | 0 (0) |
| CRT_DiCART_ on knee, first measure (14) | 0 (0) |
| CRT_DiCART_ on knee, second measure (14) | 0 (0) |
| CRT_DiCART_ on knee, third measure (14) | 0 (0) |
| CRT_DiCART_ on knee, mean (14) | 0 (0) |
| CRT_CLIN_ on chest, first measure (23) | 0 (0) |
| CRT_CLIN_ on chest, second measure (23) | 0 (0) |
| CRT_CLIN on_ chest, third measure (23) | 0 (0) |
| CRT_CLIN on_ chest, mean (23) | 0 (0) |
| CRT_DiCART_ on chest, first measure (23) | 0 (0) |
| CRT_DiCART_ on chest, second measure (23) | 0 (0) |
| CRT_DiCART_ on chest, third measure (23) | 0 (0) |
| CRT_DiCART_ on chest, mean (23) | 0 (0) |

*CRT_DiCART_ : capillary refill time measured with the DiCART™, CRT_CLIN_ : capillary refill time measured clinically*

Figure 1: plots testing the assumptions of the Bland-Altman analysis.


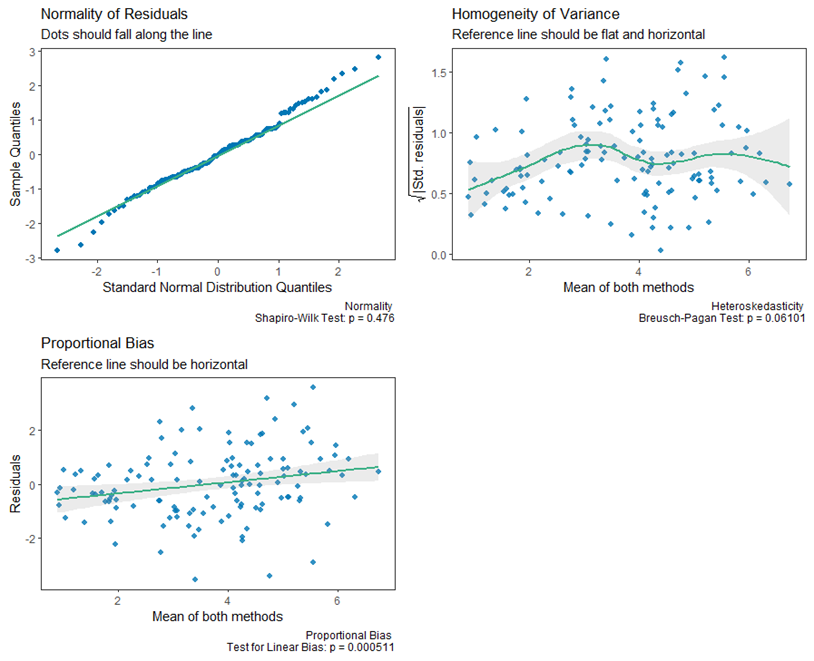

Supplement: Supplementary file 1 — Supplementary Material 1 [file 10877_2025_1271_MOESM1_ESM.docx]
